# Supplementary material for: Transcriptomic, Physiological, and Metabolomic Response of an Alpine Plant, Rhododendron delavayi, to Waterlogging Stress and Post-Waterlogging Recovery
Source: Int J Mol Sci. 2023 Jun 22;24(13):10509. doi: 10.3390/ijms241310509 (PMC10341954; doi:10.3390/ijms241310509)
Supplement: Supplementary file 1 [file ijms-24-10509-s001.zip › Supplementary Figure.pdf]

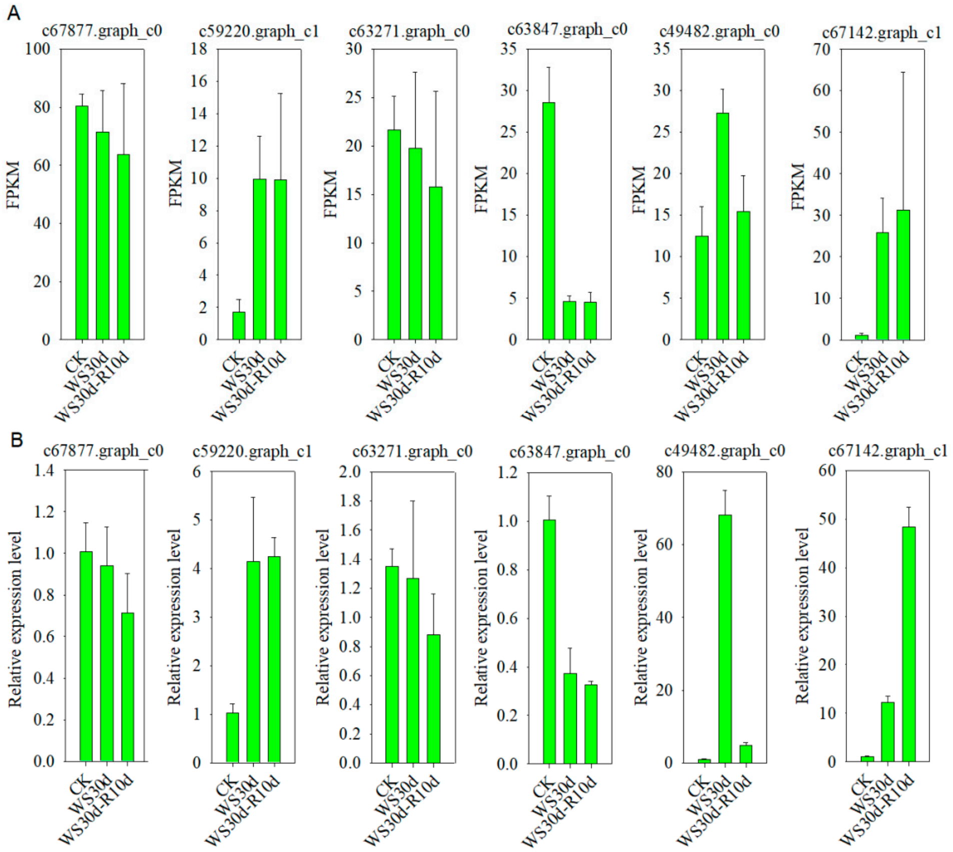

Supplementary Figure S1. Validation of putative unigenes from RNA-Seq results using qRT-PCR. (A) FPKM values of the selected genes were analyzed in CK, WS30d and WS30d-R10d leaves. (B) The relative expression levels of six DEGs used in this study were detected using qRT-PCR in in CK, WS30d and WS30d-R10d leaves. The relative expression levels of the selected genes normalized to the expression level of *β-actin* gene were calculated from cycle threshold values using the $2^{-\Delta\Delta CT}$ method. Data are mean ± standard deviation (SD) of three replicates.

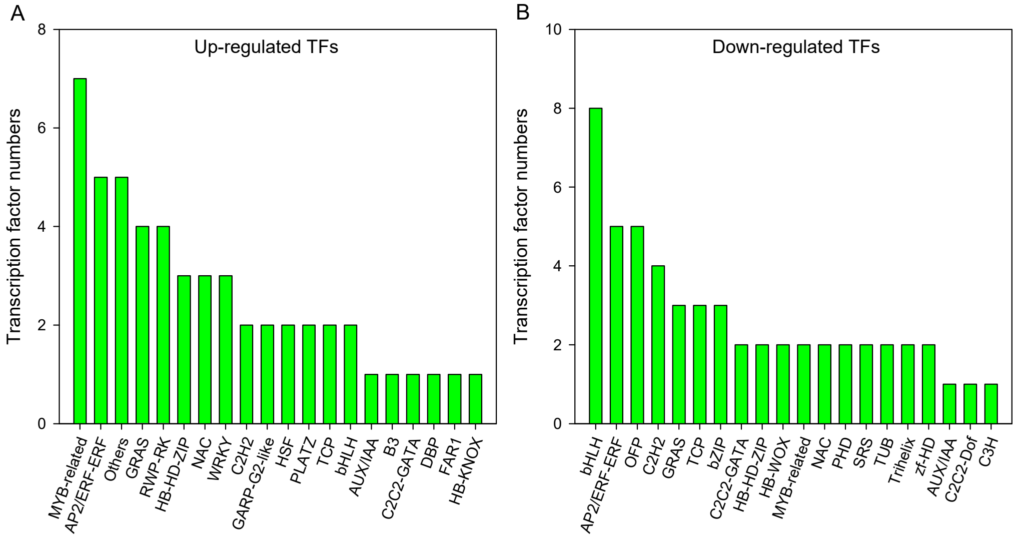

Supplementary Figure S2. The predicting transcription factors (TFs) encoded by common DEGs in *R. delavayi* leaves between WS30d and WS30d-R10d. **(A)** the TFs encoded by up-regulated DEGs; **(B)** the TFs encoded by down-regulated DEGs.

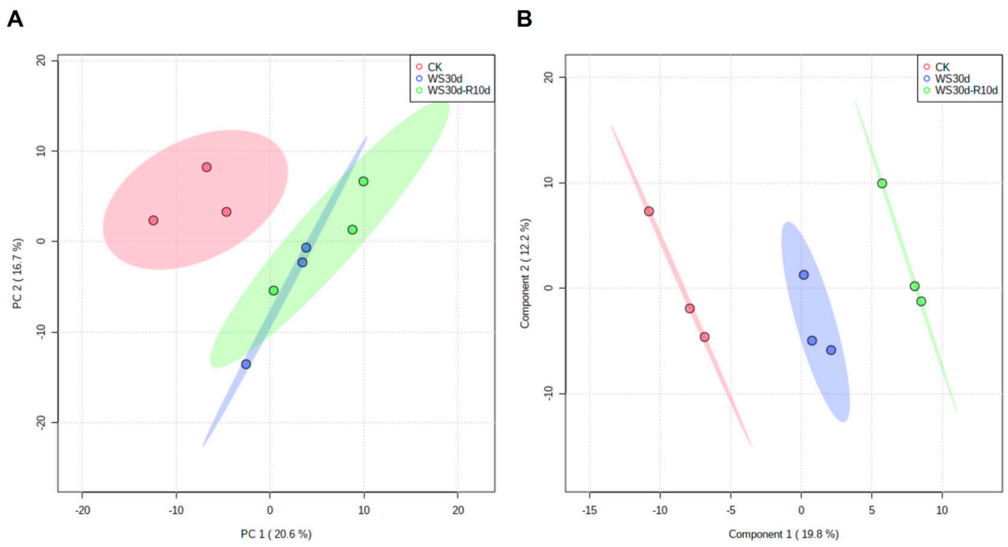

Supplementary Figure S3. The data analysis of the metabolites based on GC-TOF-MS in the CK, WS30d and WS30d-R10d. **(A)** principal component analysis (PCA); **(B)** orthogonal projections to latent structures discriminant analysis (OPLS-DA). The red, blue and green circles display 95% confidence regions of CK, WS30d and WS30d-R10d samples.
